# Mental imagery as a “*motivational amplifier*” to promote activities

**DOI:** 10.1016/j.brat.2019.02.002

**Published:** 2019-03

**Authors:** Fritz Renner, Fionnuala C. Murphy, Julie L. Ji, Tom Manly, Emily A. Holmes

**Affiliations:** a Department of Clinical Psychology and Psychotherapy, University of Freiburg, Germany; b MRC Cognition and Brain Sciences Unit, University of Cambridge, Cambridge, UK; c School of Psychological Science, University of Western Australia, Australia; d Department of Psychology, Uppsala University, Sweden

**Keywords:** Mental imagery, Mental simulation, Activity scheduling, Behavioural activation

## Abstract

Facilitating engagement in rewarding activities is a key treatment target in depression. Mental imagery can increase engagement in planned behaviours, potentially due to its special role in representing emotionally salient experiences. The present study tested the hypothesis that mental imagery promotes motivation and engagement when planning pleasant and rewarding activities. Participants were recruited from a community volunteer panel (*N* = 72). They self-nominated six activities to complete over the following week, and were randomized to either: a) a single-session *Motivational Imagery* condition (*N* = 24); b) an *Activity Reminder* control condition (*N* = 24); or c) a *No-Reminder* control condition (*N* = 24). As predicted, relative to control groups, the Motivational Imagery group reported higher levels of *motivation*, *anticipated pleasure,* and *anticipated reward* for the planned activities. The *Motivational Imagery* group also completed significantly more activities than the *Activity Reminder* group, but not more than the *No-Reminder* group. Relevance of results to behavioural activation approaches for depression are discussed.

## Highlights

• Mental imagery of planned activities increases motivation to engage in them.
• Higher imagery vividness is associated with higher anticipated reward.
• Mental imagery of planned activities is associated with higher activity completion.

Motivating people to engage in specific behaviours (e.g. exercising, healthy eating) presents a significant challenge to different areas of science concerned with human behaviour, including public health, psychology and mental health science. For many activities it is not that they are difficult to do, but they are “put off”. Behaviour change interventions have often focussed on providing people with information about the risks and benefits of various alternatives. The hope that rational ‘cognitive’ analysis of such information will significantly influence subsequent choices is, sadly, all too often misplaced (Marteau, Hollands, & Fletcher, 2012). A complementary, more emotional than cognitive-rational, route to behaviour change might be to encourage individuals to “pre-experience” future planned behaviours and the accompanying emotional consequences via mental imagery (Holmes, Blackwell, Burnett Heyes, Renner, & Raes, 2016). For example, it has been argued that imagery-based interventions might have greater impact on maladaptive motivation across a range of psychological disorders and maladaptive behaviours (e.g., Kavanagh, Andrade, May, & Connor, 2014; May, Andrade, & Kavanagh, 2015; Solbrig et al., 2018). Here we used an experimental psychopathology approach to test if motivation and engagement in both enjoyable (e.g. soaking in the bath) and routine (e.g. sorting household paperwork) activities could be facilitated by encouraging volunteers to engage in vivid mental imagery simulations of engaging in such activities.

If effective, this could be highly relevant to mental health conditions that exert a substantial burden on individuals, families and wider society. In depression, for example, individuals may experience difficulty in imagining positive aspects of future experiences and, as a consequence, be less likely to plan and engage in these activities (Holmes et al., 2016; Renner & Holmes, 2018). They can therefore deprive themselves of the potentially positive aspects (e.g. social contact) that could have improved mood symptoms. Low mood is therefore perpetuated, and negative world views remain unchallenged, due to a lack of rewarding interactions with one's environment (Lewinsohn, 1974). It is increasingly acknowledged that features such as avoidance are not unique to particular psychological disorders but part of many and, accordingly, interventions that target such features may have ‘transdiagnostic’ applications. Alterations in motivational processes such as reward anticipation are among the most common symptoms in poor mental health (Zald & Treadway, 2017) and therefore provide a candidate mechanism to target transdiagnostically. Thus, here we propose that one way to more directly target motivational processes of behavioural activities might be by simulating engagement in these planned activities via mental imagery (Renner & Holmes, 2018; Renner, Ji, Pictet, Holmes, & Blackwell, 2017).

Mental imagery refers to perceptual experiences in the absence of sensory input, regarded as a “weak” form of perception (Pearson, Naselaris, Holmes, & Kosslyn, 2015). Neuroscientific evidence suggests that the brain structures that underlie mental imagery resemble those that underlie actual perception (Kosslyn, Ganis, & Thompson, 2001; Pearson et al., 2015), giving rise to an ‘as-real’ experience of mental imagery simulations (Ji, Burnett Heyes, MacLeod, & Holmes, 2016). Recruiting mental imagery has a more powerful impact on emotions (positive and negative) than does verbal processing of the same information (Holmes, Lang, & Shah, 2009; Holmes & Mathews, 2005) and is experienced as more “real” than verbal processing (Mathews, Ridgeway, & Holmes, 2013). Vividness of mental imagery is an important aspect for the beneficial effects of imagery interventions (Blackwell et al., 2015; Renner et al., 2017). Mental imagery allows us to “pre-experience” future activities and thereby to anticipate their potential to be pleasant and rewarding (Holmes et al., 2016). Anticipating the emotional consequences of our future behaviour, whether positive or negative, is essential to decision making (Schacter et al., 2012) and guides behaviour (Sandberg & Conner, 2008). Motivation refers to an internal state that prompts and sustains goal directed behaviour. Based on previous findings, we propose that mental imagery may act as a ‘*motivational amplifier*’, promoting actual engagement in activities (Renner & Holmes, 2018) by a) increasing motivational aspects of planned activities such as anticipated reward (near transfer), which serves to b) increase behavioural engagement in planned activities (far transfer).

## 1 The present study

To test the “motivational amplifier” hypotheses, the present study tested the impact of guided motivational imagery of planned pleasant and routine activities on ratings of motivation, anticipated pleasure, anticipated reward and behavioural engagement. The motivational imagery group was compared against an activity reminder control condition as well as a no-imagery no-reminder control condition. Completion of target activities were measured over the subsequent week via an activity diary.

We hypothesized that 1) compared to the control conditions, individuals in the motivational imagery condition would show greater increases in self-ratings (motivation, anticipated pleasure and anticipated reward) of their activities from pre-to post-experimental manipulation (i.e. near transfer); 2) within the motivational imagery condition, vividness ratings of the imagined target activities would correlate positively with activity self-ratings post activity scheduling (motivation, anticipated pleasure and anticipated reward); 3) compared to both control conditions (activity reminder and no-imagery no-reminder), participants in the motivational imagery condition would engage in a higher number of scheduled target activities outside of the laboratory during participants’ everyday lives (i.e. far transfer). In addition to these a priori hypotheses, we explored a) baseline predictors of experimental effects, b) change in questionnaires measures of overall behavioural activation, depression, anxiety and stress; and c) group differences in experienced pleasure, reward, effort, and mood.

## 2 Method

### 2.1 Design

**Fig. 1:**
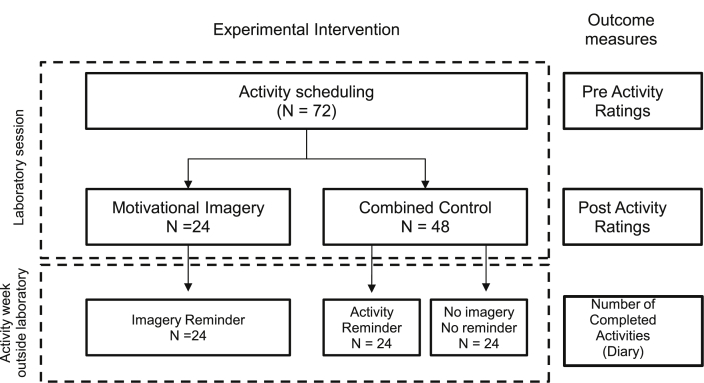
Overview of the research design, experimental conditions and outcome measures. The study consisted of two phases: Laboratory session and activity week outside laboratory. The laboratory session used a mixed design with a 2 level between-subject factor: experimental condition (motivational imagery condition; *N* = 24 vs. combined control condition; *N* = 48) and a 2 level within-subjects factor: activity ratings (pre/post activity scheduling). For the activity week outside the laboratory a three level between-subjects design was used: experimental condition (motivational imagery condition; *N* = 24 vs. activity reminder condition; *N* = 24 vs. no-imagery no-reminder control condition *N* = 24).

The study consisted of two parts: a laboratory session and an activity week during participants’ everyday lives (see Fig. 1). The laboratory session employed a mixed design with ratings completed at two points (pre activity scheduling, post activity scheduling). Activity scheduling here refers to the planning of activities. In the laboratory session, participants were randomly assigned to activity scheduling with or without motivational imagery (motivational imagery condition vs. combined control condition). For the activity week outside the laboratory, control condition participants were further assigned to either the activity reminder control condition or the no-imagery no-reminder control condition (see Fig. 1).

### 2.2 Participants

Participants (*N* = 72, 44 females, mean age: 36.35, *SD* = 16.16) were recruited from a community volunteer panel (see Table 1 for participant characteristics). Sample size was determined by considering the following parameters α = 0.05, Power = 0.90 to detect medium effects (η2 = 0.04–0.06) in a 2 × 2 repeated measures ANOVA. Inclusion criteria were: age 18–65 years; English language fluency. Exclusion criterion was current treatment for a mental health condition. The study was approved by the University of Cambridge Psychology Research Ethics Committee (PRE.2016.080).

**Table 1:** Participant characteristics.

	Condition^a^			
	Motivational Imagery (n = 24)	Activity Reminder (n = 24)	No-Imagery No-Reminder (n = 24)	ANOVA/χ^2^
**Demographic characteristics**				
Age, M (SD)	40.38 (17.94)	33.54 (15.80)	35.13 (14.16)	*F*(2,69) = 1.18, *p* = .31
Gender, n (%)				χ^2^(2,*n* = 72) = 3.19, *p* = .20
Female	18 (75)	18 (75)	13 (54)	
Male	6 (25)	6 (25)	11 (46)	
Years of education, M (SD)	16.42 (3.30)	17.88 (3.13)	17.04 (3.43)	*F*(2,69) = 1.19, *p* = .31
Occupation, n (%)				χ^2^(6,*n* = 72) = 2.41, *p* = .88
Employed FT	3 (12.5)	5 (20.8)	5 (20.8)	
Employed PT	5 (20.8)	5 (20.8)	4 (16.7)	
Unemployed	3 (12.5)	1 (4.2)	1 (4.2)	
Student	13 (54.2)	13 (54.2)	14 (58.3)	
Marital status, n (%)				χ^2^(6,*n* = 72) = 8.38, *p* = .21
Single	5 (20.8)	10 (41.7)	12 (50)	
In relationship	9 (37.5)	8 (33.3)	3 (12.5)	
Married	9 (37.5)	5 (20.8)	9 (37.5)	
Divorced	1 (4.2)	1 (4.2)	–	

a Statistics reported in this Table tested differences between 3 conditions (motivational imagery, activity reminder, no-imagery no-reminder). Additional comparison between the motivational imagery condition and the combined control condition yield no significant group differences at baseline.

### 2.3 Materials

#### 2.3.1 Baseline questionnaires

*Spontaneous Use of Imagery Scale (SUIS)* (Reisberg, Pearson, & Kosslyn, 2003) – Twelve statements assess use of imagery in everyday life on a five-point scale ranging from 1 “never” to 5 “always”. An example item is: “When I think about a series of errands I must do, I visualize the stores I will visit”.

*Plymouth Sensory Imagery Questionnaire (PSI-Q)* (Andrade, May, Deeprose, Baugh, & Ganis, 2014) – Thirty-five items assess vividness of mental imagery in different modalities (vision, sound, smell, taste, touch, bodily sensations, emotional feelings) on a scale ranging from 0 “no image at all” to 10 “image as clear and vivid as real life”. An example item is: “Imagine the appearance of a friend you know well”.

*Depression Anxiety and Stress Scale (DASS-21)* (Lovibond & Lovibond, 1995) – Twenty-one statements rated on a four-point scale ranging from 0 “did not apply to me at all” to 3 “applied to me very much or most of the time”. The scale assesses depression, anxiety, and stress over the past week.

*Positive and Negative Affect Scale State Version (PANAS)* (Watson, Clark, & Carey, 1988) – Ten adjectives assessing current positive affect and ten adjectives assessing current negative affect rated on a scale ranging from 1 “not at all” to 5 “extremely”.

*Behavioural Activation for Depression Scale (BADS)* (Kanter, Mulick, Busch, Berlin, & Martell, 2007) – Twenty-five item scale assessing levels of behavioural activation in the past week on a scale ranging from 0 “not at all” to 6 “completely”. An example item is: “I engaged in a wide and diverse array of activities”.

*Temporal Experience of Pleasure Scale (TEPS)* (Gard, Germans Gard, Kring, & John, 2006) – Eighteen item scale assessing individual trait dispositions in anticipatory and consummatory experiences of pleasure on a scale ranging from 1 “very false for me” to 6 “very true for me”. An example item is: “When something exciting is coming up in my life, I really look forward to it”.

*Dimensional Anhedonia Rating Scale (DARS)* (Rizvi et al., 2015) – A self-report scale assessing anhedonia across four domains: pastimes/hobbies, food/drinks, social activities, and sensory experiences. For each domain participants are required to write down two examples of what they find rewarding. Participants then provide ratings of desire, motivation, effort and consummatory pleasure for the examples provided.

##### 2.3.1.1 Identifying target activities

Participants identified six activities they would like to engage in over the subsequent week from a pre-defined list of broader activity categories (see Supplementary Table 1): three enjoyable activities, i.e. activities that they would enjoy but seldom got around to doing and three routine activities, i.e. activities that they had been putting off doing but would find satisfying and rewarding once completed. They were instructed to select only activities they did not already routinely engage in, that did not require extensive preparation, and that could be done for 10 min or longer. Examples of selected activities include “reading before bed” “doing yoga exercises” “working on a crossword” or “clearing out old items”. Where the activity chosen by a participant was broad (e.g. “research how to do something”), s/he was asked to choose a more specific activity (e.g. “research different energy supplier options”).

##### 2.3.1.2 Activity ratings

Before the experimental manipulation, participants rated their *motivation, anticipated pleasure, anticipated reward, and anticipated effort* for each of the target activities. Ratings were made for each of the six scheduled target activities pre and post activity scheduling on 100 mm Visual Analogue Scales (VAS) with the endpoints “*not at all”* to “*extremely”*. An example item illustrated how to use the rating scale. Participants did not receive a formal definition of the target constructs but had the opportunity to ask questions. The motivational imagery group repeated these ratings directly after activity scheduling with imagery simulation of the respective activity. Participants in the activity reminder control group and participants in the no-imagery no-reminder control group repeated these ratings directly after activity scheduling (with no imagery instructions of the respective activity). Prior to activity scheduling, participants also rated how important each selected activity was to them, how much they had been putting each activity off, and how difficult they typically found it to make time for the activity. These ratings were also made on VAS with the endpoints “*not at all”* to “*extremely”*.

##### 2.3.1.3 Mental imagery vividness ratings

Participants in the motivational imagery condition rated the vividness of their mental imagery immediately following mental imagery of their target activities, on a 100 mm VAS asking “How vividly were you able to imagine the activity?” with response anchors ranging from 0 “not at all” to 100 “extremely”.

##### 2.3.1.4 Activity scheduling

Participants then scheduled the selected activities in over the course of the following week using an activity diary and following the procedures and principles described in Behavioural Activation treatment manuals (Martell, Dimidjian, & Herman-Dunn, 2010, p. 89–108). The experimenter and participant specified what the participant would do in behavioural terms (e.g. tidy my desk), where and when the activity would occur (e.g. in my office on Saturday morning) and the context in which the activity would take place (e.g. after breakfast).

Participants in the motivational imagery condition (n = 24) then completed a general exercise in generating vivid mental imagery (Holmes & Mathews, 2005) and received instructions to practice doing each activity, using mental imagery, as if seeing and experiencing it, so they were actively involved in the activity in their mind's eye. They were then guided through an imagery script, separately for each of their six selected activities, as described below.

##### 2.3.1.5 Motivational mental imagery of target activities

The imagery script requests participants to vividly imagine themselves performing each of their six target activities, with each resolving in a positive way (see supplementary materials). Participants were instructed to concentrate and close their eyes during the imagery task and to focus on the most positive aspects of their image while imagining themselves doing the activity. The experimenter guided participants through an imagery script focussing on 1) contextual cues of the activity (e.g. date/time, context and place) as specified by participants at the time of scheduling the activities; 2) multi-sensory (e.g. visual, auditory, sensory) engagement in the activity; 3) positive aspects associated with the activity; and 4) vivid imagery of the most powerful and motivating part of their image. The script was written by the research team following initial pilot testing. During the experimental session the experimenter read the script to the participant inserting the relevant information for the respective activity (context, time, date etc.) while reading the script out loud. Each imagery-activity script took about 1–2 min.

##### 2.3.1.6 Reminder messages

Participants in the motivational imagery condition received text messages during the activity week to facilitate transfer from the experimental manipulation during the laboratory session to the activity week outside the laboratory. These reminder prompts also, of course, acted as a reminder of those activities. To control for the presentation of reminders per se, participants in the activity-reminder control condition also received text messages reminding them of the planned activities, but without imagery instructions. Participants in both the motivational imagery condition and activity reminder condition received two text messages per day during the activity week outside the laboratory. The text messages did not specify any detail about the nature or timing of the upcoming activity. In the motivational imagery condition the text message encouraged use of vivid mental imagery of the most powerful and motivating part of their image for the upcoming activity. In the activity reminder condition the text message included the words “activity reminder” to remind participants of the upcoming activity.

## 3 Procedure

Participants completed self-report questionnaires (Table 2) after providing written and informed consent. Next, participants identified their “enjoyable” and *“*routine*”* target activities (akin to standard procedures for Behavioural Activation treatment), and provided activity ratings for each activity and scheduled them for the next week. The activity scheduling was done with or without mental imagery (motivational imagery condition vs. control conditions). Participants were then given an activity diary with instructions on how to record the following after each completed activity each day: (1) whether or not they had completed their planned activity, including when and for how long, (2) mood ratings immediately following each activity, and (3) where they had not managed to complete an activity, to provide a reason why. Participants in the motivational imagery condition and in the activity reminder condition were then informed that they would receive two text messages at random points in time before each planned activity (see above). At the end of the session, participants received payment for their time and a pre-paid return envelope to post the activity diary back.

**Table 2:** Baseline questionnaires and baseline activity ratings per condition.

Baseline Questionnaires	Condition^a^			
	Motivational Imagery (n = 24)	Activity Reminder (n = 24)	No-Imagery No-Reminder (n = 24)	ANOVA/χ^2^
Imagery				
SUIS, M (SD)	36.83 (7.40)	35.88 (7.84)	40.45 (6.80)	*F*(2,69) = 2.59, *p* = .08
PSI-Q	6.96 (1.30)	6.95 (1.38)	6.79 (1.55)	*F*(2,69) = 0.11, *p* = .89
Mood				
Stress (DASS-21)	4.58 (3.15)	4.54 (4.35)	4.20 (2.62)	*F*(2,69) = 0.09, *p* = .92
Anxiety (DASS-21)	1.5 (2.46)	2.38 (3.29)	1.88 (2.44)	*F*(2,69) = 0.62, *p* = .54
Depression (DASS-21)	2.04 (2.46)	3.75 (5.35)	2.00 (2.21)	*F*(2,69) = 1.18, *p* = .17
Positive Affect	29.42 (5.71)	30.71 (7.88)	31.08 (6.87)	*F*(2,69) = 0.39, *p* = .68
Negative Affect	11.79 (2.19)	12.46 (4.19)	12.25 (3.01)	*F*(2,69) = 0.27, *p* = .77
Behavioural Activation				
BADS Total	112.79 (23.34)	103.17 (26.35)	107.08 (21.54)	*F*(2,69) = 0.99, *p* = .38
Pleasure/Anhedonia				
TEPS Anticipatory	36.83 (5.14)	36.25 (5.24)	36.92 (5.59)	*F*(2,69) = 0.11, *p* = .90
TEPS Consummatory	42.38 (5.69)	41.63 (5.50)	44.42 (5.60)	*F*(2,69) = 1.60, *p* = .21
DARS	81.21 (11.73)	82.38 (10.25)	84.29 (12.30)	*F*(2,69) = 0.44, *p* = .64
**Baseline activity ratings**				
Putting off activity	69.79 (14.12)	68.92 (11.81)	69.97 (21.61)	*F*(2,69) = 0.03, *p* = .97
Importance of activity	66.05 (12.47)	74.02 (12.51)	69.32 (14.34)	*F*(2,69) = 2.23, *p* = .12
Difficulty to make time for activity	59.83 (22.75)	60.17 (21.75)	63.85 (21.20)	*F*(2,69) = 0.25, *p* = .78

**Note.** SUIS = Spontaneous Use of Imagery Scale, PSI-Q = Plymouth Sensory Imagery Questionnaire, DASS-21 = Depression, Anxiety and Stress Scale, BADS = Behavioural Activation for Depression Scale, TEPS = Temporal Experience of Pleasure Scale, DARS = Dimensional Anhedonia Rating Scale.

a Statistics reported in this Table tested differences between three conditions (motivational imagery, activity reminder, no-imagery no-reminder). Additional comparisons between the motivational imagery condition and the combined control condition yield no significant group differences at baseline.

## 4 Statistical analyses

All statistical tests were two-tailed (significance level alpha .05). Significant omnibus tests were followed up by Bonferroni corrected pairwise comparisons. Effect sizes (η^2^ and Cohen's *d*) were calculated for analyses involving group comparisons.

### 4.1 Laboratory session, hypothesis 1 and hypothesis 2

There were three conditions 1) motivational imagery 2) activity reminders control and 3) no-imagery no-reminders control. For the purposes of examining the effects of the laboratory session on ratings, conditions 2 and 3 (which were at this stage identical) were collapsed into a combined control condition (see Fig. 1). To test differences between the motivational imagery and combined control condition in activity ratings from pre to post activity scheduling (hypothesis 1), a repeated-measures ANOVA was conducted for each activity rating, with condition (motivational imagery vs. combined-control) as the between-subject factor and time (pre vs. post activity scheduling) as the within-subject factor. Vividness ratings were correlated with the post activity scheduling activity ratings in the motivational imagery group (hypothesis 2).

### 4.2 Behavioural activity outcomes, hypothesis 3

To test differences between the three conditions in the number of completed activities (hypothesis 3), a one-way ANOVA was conducted, with condition (motivational imagery, activity reminder, no-imagery no-reminder) as the between-subjects factor and the number of completed activities as the dependent variable.

### 4.3 Exploratory post-hoc analyses of activity ratings and mood questionnaires

Additional exploratory analyses focussed on differences between conditions on overall behavioural activation, stress, anxiety and depression ratings from pre to post activity week using repeated-measures ANOVA with condition (motivational imagery vs activity reminder vs no-imagery no-reminder) as the between-subjects factor and time (baseline, post activity week) as the within-subjects factor.

To explore psychological mechanisms of motivational imagery effects, activity ratings collected following activity selection were entered in a multiple regression analysis predicting the number of completed activities. Condition was first dummy coded with the motivational imagery condition as reference group and two dummy variables (one representing motivational imagery vs. activity reminder condition and one representing motivational imagery vs no-imagery no-reminder condition) were entered in a linear regression model. The different activity ratings were entered in separate regression models (one for each activity rating). An interaction term between the activity ratings and the dummy variables was added to test whether the different activity ratings differentially predicted the number of completed activities by condition.

## 5 Results

### 5.1 Baseline ratings of selected activities and baseline questionnaires

Comparing baseline activity ratings and scores on baseline questionnaire measures (i.e. prior to the experimental manipulation) confirmed no significant differences between the groups prior to the experimental manipulation (Table 2). Scores on scales assessing depression and anxiety (DASS-21) were low on average, with little variability, and in the normal mood range according to established cutoffs (Lovibond & Lovibond, 1995).

### 5.2 Manipulation check

All participants were able to identify six behavioural target activities (three enjoyable and three routine activities) and to plan these in their activity diary. At baseline the three selected “enjoyable” activities were rated as more enjoyable (*M* = 78.63, *SD* = 12.35) than the three selected “routine” activities (*M* = 36.82, *SD* = 18.05; *t*(71) = 16.57, *p* < .001, *d* = 2.70). Within the motivational imagery condition, vividness ratings during the laboratory session were high (*M* = 79.41, *SD* = 14.56 on a scale ranging from 0 = not vivid at all to 100 = extremely vivid), indicating that participants in the motivational imagery condition were able to form vivid images of engaging in their selected activities. For each activity, we asked participants in the motivational imagery condition to indicate if they used the reminder prompt to engage in imagery of that activity. Possible answers: yes, 1x; yes, 2x; no. On average, for 12 reminder prompts in the motivational imagery condition, participants indicated that they imagined engaging in the activity approximately half of the time they received a reminder prompt (*M* = 6.22, *SD* = 4.03). Participants in the activity reminder condition were asked, for each activity if the reminder messages helped to remind them of the planned activities: Possible answers: Yes/No. On average, for six planned activities (12 reminder prompts), participants in the activity reminder condition indicated that the messages helped them to remind them of the activities for approximately two of the six planned activities (*M* = 2.19, *SD* = 1.86).

### 5.3 Motivation and anticipatory reward

**Fig. 2:**
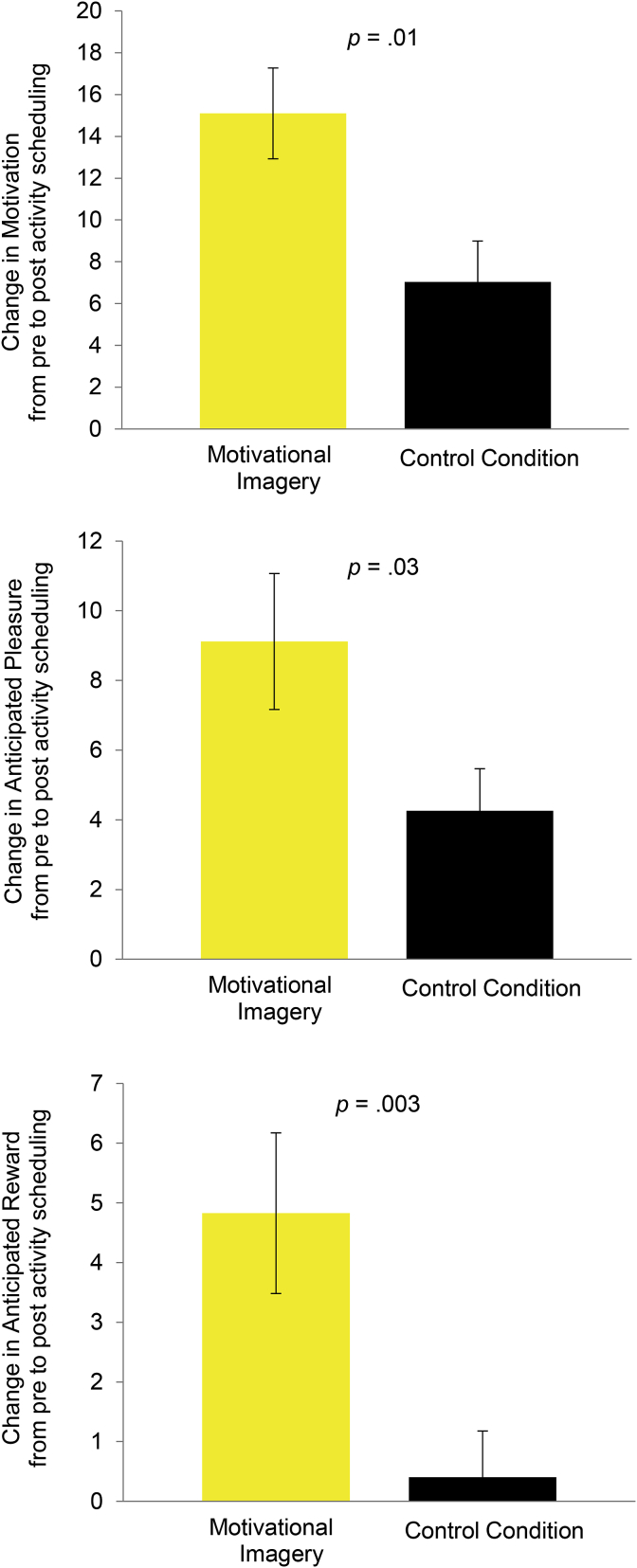
Change in motivation, anticipated pleasure and anticipated reward ratings of activities from pre activity scheduling to post activity scheduling for the motivational imagery condition (*n* = 24) in yellow and the combined-control group (*n* = 48) in black. The bars show the observed mean change score and the error bars the standard error of the mean. (For interpretation of the references to colour in this figure legend, the reader is referred to the Web version of this article.)

*Ratings of activities pre and post activity scheduling (hypothesis 1)*. Activity ratings before and after the experimental manipulation separately for the three groups are summarized in Table 3. There was a significant Condition × Time interaction for motivation, *F* (1, 70) = 6.47, *p* = .01, η^2^ = 0.085, anticipated pleasure, *F* (1,70) = 4.91, *p* = .03, η^2^ = 0.066, and for anticipated reward, *F*(1,70) = 9.31, *p* = .003, η^2^ = 0.117^2^. As predicted, the increase in activity ratings was significantly larger in the motivational imagery condition than in the combined control condition (Fig. 2). Anticipated effort from engaging in the scheduled activities decreased in both conditions from pre to post activity scheduling. The Condition × Time interaction for anticipated effort was not significant, *F*(1,70) = 2.61, *p* = .11, η^2^ = 0.036. For separate analyses of routine and enjoyable activities see Supplementary Materials.

**Table 3:** Activity ratings before and after the experimental manipulation.

	Before Activity Scheduling		After Activity Scheduling	
	Motivational Imagery (n = 24)	Combined Control (n = 48)	Motivational Imagery (n = 24)	Combined Control (n = 48)
Motivation	55.92 (14.25)	60.46 (15.39)	71.02 (11.00)	67.50 (13.98)
Anticipated pleasure	55.14 (10.58)	59.01 (11.33)	64.26 (12.85)	63.27 (12.13)
Anticipated reward	77.21 (9.72)	80.26 (8.75)	82.03 (8.90)	80.66 (10.98)
Anticipated effort	58.24 (11.73)	57.02 (16.03)	51.90 (13.51)	56.17 (16.81)

*Relation between imagery vividness and activity ratings post activity scheduling (hypothesis 2).* As predicted, there was a significant positive correlation between vividness and anticipated reward ratings, (*r* = 0.48, *p* = .02); the more vividly participants imagined engaging in the scheduled activities, the higher their anticipated reward of engaging in them. There were no significant correlations between vividness and anticipated pleasure (*r* = 0.38, p = .08) or between vividness and motivation (*r* = 0.24, *p* = .28).

### 5.4 Behavioural engagement in planned activities

*Effects of motivational imagery on the number of completed activities (hypothesis 3).* The three groups differed in the mean number of completed activities *F*(2,67) = 3.71, *p* = .03, *d* = 0.74. In line with our hypothesis, post-hoc comparisons with Bonferroni correction for multiple testing revealed a higher completion rate of activities in the motivational imagery condition (*M* = 4.78, *SD* = 0.90) compared to the activity reminder control condition (*M* = 3.83, *SD* = 1.11), *p* = .033, *d* = 0.94); that is, imagery had an effect over and above simple reminders. There was no statistical significant difference in activity completion rate between the motivational imagery condition and the no-imagery no-reminder control condition (*M* = 4.54, *SD* = 1.59), *p* = 1.00, *d* = 0.19; imagery did not have an effect above the no-imagery no-reminder control condition. There was no statistical significant difference in activity completion rate between the two control conditions, *p* = .16, *d* = 0.52; suggesting text reminders in themselves were not linked with improved completion. For separate analyses of routine and enjoyable activities see Supplementary Materials.

### 5.5 Exploratory post-hoc analyses

#### 5.5.1 Baseline predictors of motivational imagery effects

**Fig. 3:**
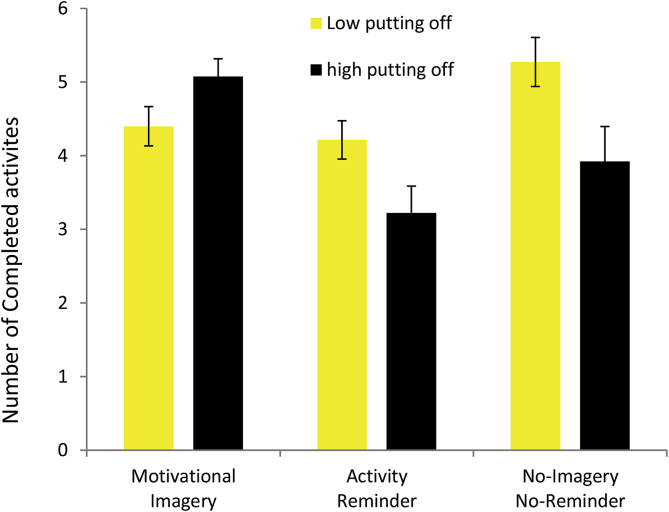
Illustration of the number of completed activities per condition according to whether the participant had formerly “put off” doing that particular activity (as rated at baseline). This indicates that for those activities that had been previously more put off participants were more likely to benefit from the imagery intervention.

When selecting activities, participants were asked to rate how much they had formerly “put off” (delayed or procrastinated in completing) these activities. There was a significant interaction between “putting off” ratings and the dummy variable comparing activity completion between the motivational imagery condition and the activity reminder condition, *β* = −1.79, *t*(64) = −2.54, *p* = .01 and for “putting off” ratings and the dummy variable comparing activity completion between the motivational imagery condition and the no-imagery no-reminder condition, *β* = −1.71, *t*(64) = −2.94, *p* = .005. To interpret the interaction we plotted the number of completed activities as a function of “putting off ratings” (low/high median split) for each condition (Fig. 3). This indicates that for those activities that were put off more participants were most likely to benefit from the imagery intervention whereas this pattern was reversed in the control conditions. The remaining activity ratings did not predict the number of completed activities (all p-values >.05).

#### 5.5.2 Changes in overall behavioural activation and stress, anxiety, depression ratings from baseline to post activity week

We explored if a broader increase in self-reported behavioural activation, assessed with the BADS, and scores on the depression, anxiety, and stress scales of the DASS-21 changed from baseline to post activity week. Overall there was a significant increase in behavioural activation from baseline (*M* = 106.84, *SD* = 24.44) to post activity week (*M* = 113.90, *SD* = 21.66), *F*(1,64) = 9.76, *p* = .003, *η*^2^ = 0.132. The interaction with condition was not significant, indicating that the increase in behavioural activation as indexed by the BADS from baseline to post activity week did not differ between the three groups, *F*(2,64) = 0.02, *p* = .977, *η*^*2*^ = 0.001.

Overall there was a decrease on the stress scale of the DASS-21 from baseline (*M* = 4.46, *SD* = 3.43) to post activity week (*M* = 3.63, *SD* = 3.37), *F*(1,64) = 6.25, *p* = .015, *η*^*2*^ = 0.089. The interaction with condition was not significant, indicating that the decrease in stress ratings from baseline to post activity week did not differ between the three groups, *F*(2,64) = 0.74, *p* = .48, *η*^*2*^ = 0.023. Anxiety ratings on the DASS-21 did not change over the study period, *F*(1,64) = 0.11, *p* = .736, *η*^*2*^ = 0.002 and neither did depression ratings, *F*(1,64) = 0.39, *p* = .535, *η*^*2*^ = 0.006.

#### 5.5.3 Mood and activity rating upon activity completion

Overall, completed activities were rated as pleasant (*M* = 65.48, *SD* = 14.18) and rewarding (*M* = 77.57, *SD* = 11.10) with no differences between the three groups, *F*(2,64) = 0.81 *p* = .45, *η*^*2*^ = 0.025 and *F*(2,64) = 0.78 *p* = .46, *η*^2^ = 0.024, respectively. Average effort ratings of completed activities were around the centre of the VAS scale (*M* = 46.41, *SD* = 15.11) with no differences between the three groups, *F*(2,64) = 0.49 *p* = .61, *η*^2^ = 0.015. Participants reported positive mood during engagement in the completed activities (*M* = 70.69, *SD* = 11.97) with no differences between the three groups, *F*(2,64) = 0.21 *p* = .81, *η*^2^ = 0.007.

## 6 Discussion

This study examined whether guided motivational imagery enhanced motivation for, and behavioural engagement in planned enjoyable and routine activities. Results suggest mental imagery can act as a *motivational amplifier* for both planned enjoyable and routine activities. Compared with two conditions in which imagery was not explicitly encouraged, participants who had engaged in mental imagery simulation showed a stronger increase on all scales assessing motivational aspects of the scheduled activities (motivation, anticipated pleasure, and anticipated reward) from pre to post activity scheduling. In addition, despite receiving equal number of text reminders, participants encouraged to use imagery completed significantly more activities than those who were not given explicit imagery guidance.

A caveat here is that the completion rates were not significantly greater in the motivational imagery group than those of the no-imagery no-reminder control group who received neither explicit imagery guidance nor reminders. In a between-participant design there are many factors relating to individual propensity for task completion and variation in task selection. For example, while not statistically significant, participants in the no-imagery no-reminder condition had relatively high scores on the Spontaneous Use of Imagery Scale (SUIS) compared to the other two groups. While speculative, it is possible that participants in this group tended to use imagery spontaneously during the activity week. An alternative explanation is that the significant effect of group on the number of completed activities is actually driven by a low number of completed activities in the activity reminder condition. Participants in this condition might have experienced the activity reminders as irritating resulting in a lower completion rate. In line with this explanation, when asked if they found the reminder prompts useful in reminding them of the planned activities, participants in the activity reminder condition indicated they only found the prompts useful for approximately two out of six activities. Further work using a greater range of activities over a longer period and potentially more sensitive measures such as ‘experience sampling’ will help to establish the likely reliability of the enhanced completion finding. Overall, however, these findings are in line with the overarching hypothesis that using mental imagery can promote engagement in behavioural activities.

Why might mental imagery of future behavioural activities promote motivation and actual engagement? Mental imagery is a core component of the ‘prospective brain’, a functional network allowing us to predict and plan for the future (Moulton & Kosslyn, 2009; Schacter, Addis, & Buckner, 2008). Mental images can evoke strong emotional and neurophysiological reactions (Ji et al., 2016) and imagery simulations of future events impact subjective probability ratings of these events to happen (Raune, MacLeod, & Holmes, 2005). These features of mental imagery may impact motivational aspects of future behaviours such as anticipated reward and anticipated pleasure when simulating engagement in positive future events. Anticipated emotions in turn drive decision making and goal directed behaviours (Gorka et al., 2014). For example, albeit in the opposite direction, greater levels of anticipated regret of engaging in a health risk behaviour predict weaker behavioural engagement in these behaviours (Brewer, DeFrank, & Gilkey, 2016). Similarly, increasing positive anticipated emotions via mental imagery may have a knock-on effect on motivation and behaviour, i.e. act as a *motivational amplifier*. As one participant in the motivational imagery condition noted in a feedback email: “*I thought doing visualisations was a great way to get myself to do the activities I miss out on, and it does seem to work. It made me happy and look forward to doing the activities*”.

Mental imagery of future behavioural activities in this study increased anticipated pleasure and anticipated reward, relative to control groups. Thus individuals in the motivational imagery condition predicted that engaging in the planned activities would be more pleasant and more rewarding compared to those in control groups. However, ratings of actual pleasantness and reward upon activity completion did not differ between the groups, suggesting that there was a discrepancy between what participants predicted and how the planned activities actually turned out to be once completed. This finding is interesting in the context of the affective forecasting literature. When people make predictions of how they will feel about events in the future (i.e. affective forecasts) they typically overestimate the emotional impact of these events (Wilson & Gilbert, 2005). Importantly, predictions of how we will feel about events in the future drive our decision making and actions (Mellers & McGraw, 2001). In the context of depression it has been shown that affective forecasting in individuals with depression is biased towards more extreme negative affective forecasts and less extreme positive forecasts compared to healthy controls (Wenze, Gunthert, & German, 2012). Our findings suggest that the imagery manipulation increased the affective forecasting bias for positive future events. Future research should test if similar imagery interventions can help reduce the negative forecasting bias in depression.

We also found that the more vividly participants imagined the scheduled activities the higher their anticipated reward ratings of engaging with them. Future research should further explore boosting positive imagery vividness in promoting motivation for behavioural activities, specifically in individuals who struggle to form vivid positive images such as those with depression (Holmes et al., 2016).

We also explored baseline predictors of motivational imagery effects and found that the propensity of participants to “put-off” activities in the past predicted activity completion rates (Fig. 3). This suggests that the most difficult behavioural targets, things that people have been *least* inclined to do, may benefit most from this imagery intervention. This makes more intuitive sense when expressed in terms of the corollary; participants are more likely to engage in behaviours that they have not tended to “put off” thus reducing any *additional* benefit of imagery. It is nevertheless encouraging in terms of procrastination, delaying unpleasant activities despite negative consequences of such delay (Klingsieck, 2013). In a health context, procrastination has been linked to poor physical health (Sirois, 2015; Sirois, Melia-Gordon, & Pychyl, 2003) and mental health (Stead, Shanahan, & Neufeld, 2010) outcomes, including anxiety and depression. If, as our data suggest, motivational imagery interventions may be particularly effective for these types of activities the clinical implication of these results may be considerable. The results are also consistent with our previous studies showing beneficial effects of positive mental imagery on behavioural activation in individuals with depression (Renner et al., 2017).

Our findings, if replicated, have potential implications for research on novel intervention development in clinical psychology and public health. Imagery procedures could potentially be used to increase motivation and engagement in health related behaviours such as physical activities (Chan & Cameron, 2012) or healthy eating (Knäuper et al., 2011). Similarly, psychological interventions aiming to increase behavioural activation (Martell, Addis, & Jacobson, 2001; Martell et al., 2010) could potentially benefit from the motivation enhancing effects of mental imagery simulations of enjoyable and routine activities (Renner et al., 2017; Renner & Holmes, 2018). This is consistent with a mechanistic approach to intervention innovation by focussing on core brain-behaviour dimensions (Insel et al., 2010) (e.g. reward anticipation) to identify psychological mechanisms that are common to multiple mental disorders and to develop new, more direct, ways to target these (Holmes, Craske, & Graybiel, 2014; Holmes et al., 2018). However, note that the motivation enhancing effects of imagery might not transfer to a behavioural level as our data suggests that the completion rates were not significantly greater in the motivational imagery group than those of the no-imagery no-reminder control group. The findings of this study provide initial evidence suggesting that mental imagery of planned activities increases motivation to engage in these activities. This core finding now needs replication in independent samples following the same experimental procedures after appropriate training in the use of the mental imagery as motivational amplifier protocol. There are clearly several areas of the wider psychology literature that are relevant to this topic, which should also be further explored in future developments of this work including work on implementation intentions, mental contrasting, and goal and activity planning (e.g. Coote & MacLeod, 2012; Farquharson & MacLeod, 2014; Gollwitzer, 1999; Gollwitzer & Sheeran, 2006; Oettingen & Mayer, 2002, 2016).

Several limitations should be taken into account when considering the results of this study. We recruited a non-clinical sample from the general population and included a broad range of potentially enjoyable and rewarding activities. It remains unclear if our results would generalize to specific behavioural activities in target groups such as physical activities for sedentary individuals. Future studies might focus on specific target groups and specific types of behaviours. We used self-report scales to assess activity ratings in the lab. Future studies should seek to include additional measures of motivation that include objective measures to supplement behavioural findings.

In summary, the current study provides proof of concept evidence that mental imagery, by allowing individuals to pre-experience rewarding and positive aspects of potential future activities, might act as a *motivational amplifier*: Simulating engagement in future behavioural activities via mental imagery increases motivation to engage in these activities which can transfer to actual behaviour. These findings can help inform future studies that aim to develop and apply mental imagery interventions in the areas of public health and clinical psychology where the aim is to motivate people for behaviour change, and ultimately, improve people's quality of life, and wellbeing.

## Conflicts of interest

Emily Holmes is Associate Editor at Behaviour Research and Therapy. The authors declare no further competing interests.

## Data statement

Data and materials are available via the Open Science Framework and can be accessed at osf.io/x238k, or are otherwise available from the authors on reasonable request (with the exception of questionnaire measures subject to third-party copyright or potentially identifying participant information). Experimental protocols will need to be accompanied by training to use the motivational amplifier protocol.

## Footnotes

2 For motivation, two values in the control condition were >1.5 x interquartile range; For anticipated pleasure one value in the control condition was >1.5 x interquartile range; For anticipated reward one value in the motivational imagery group was >1.5 x interquartile range. Excluding these values from analyses did not change the result significance pattern.
