# Supplementary material for: Mental imagery as a “motivational amplifier” to promote activities
Source: Behav Res Ther. 2019 Mar;114:51–9. doi: 10.1016/j.brat.2019.02.002 (PMC6416378; doi:10.1016/j.brat.2019.02.002)
Supplement: Multimedia component 1 [file mmc1.docx]

**Supplementary materials**

Data and materials are available via the Open Science Framework and can be accessed at osf.io/x238k, or are otherwise available from the authors on request (with the exception of questionnaire measures subject to third-party copyright or potentially identifying participant information). Experimental protocols will need to be accompanied by training to use the motivational amplifier protocol.

## Imagery Script

“I would now like you to imagine [ACTIVITY]. Just like in the cutting the lemon example/just like with the previous activity but this time imagine [ACTIVITY] and focus on the positive aspects of [ACTIVITY].”

- Please focus on your image of the most positive aspects of [ACTIVITY]
- Please shut your eyes while imagining (no need to repeat if they already closed eyes)
- Please imagine yourself doing the activity
- Concentrate, as it is easy to slip into your normal way of thinking

---START IMAGERY SCRIPT ---

Close your eyes and relax.

---Part 1 contextual cues ---

Remember to imagine and to see, to hear and to feel yourself really there. As vividly as you can. Imagine it is [DATE/TIME]. And you are at [PLACE/SITUATION/CONTEXT]. You have planned to [ACTIVITY]. Imagine yourself in this situation. Notice your surroundings at [PLACE/SITUATION/CONTEXT] that reminds you to engage in your planned activity. Take a second to focus vividly on that ….. [PAUSE]

---Part 2 engaging in the activity ---

Now imagine that you are following your plan of [ACTIVITY]. Imagine the details, as if you are really doing it, what you can see … hear ... smell … feel … [PAUSE]

If you would like, go back and imagine it one more time to get it really right and just how you would like the activity to be …. [PAUSE]

--- Part 3 positive outcome for activities ---

| --- **for ENJOYABLE activities** ---  Now imagine how good it feels to be spending some time on [ACTIVITY]. [PAUSE] Focus on the most positive aspects.  Take your time. [PAUSE]. Experience any pleasant feelings and sensations that you associate with [ACTIVITY]. [PAUSE] Notice how good that feels. [PAUSE]. | --- **for ROUTINE activities** ---  Now imagine how good it feels to be making some progress with/completing [ACTIVITY]. Focus on the most positive aspects.  Take your time. [PAUSE]. Experience any pleasant feelings and sensations that you associate with making some progress with/completing this activity [PAUSE]. Imagine telling yourself well done. [PAUSE] |
| --- | --- |

---Part 4 snapshot activity image ---

Now focus on the part of your image that will help you most to be motivated to actually do [ACTIVITY]. Make it really vivid. As vivid and as real as you can. Well done. Try to remember this part. These are the aspects of your image I’d like you to be able to bring back to mind. Later. Mentally photograph this part of your image – take a snapshot image of the part that is most powerful/significant and that will most help to motivate you to actually do the activity. This is your ACTIVITY IMAGE. Remember that image.

Take your time and whenever you feel you are finished, you can open your eyes again.

**Supplementary results**

*Activity Ratings before and after the experimental manipulation separately for enjoyable and routine activities*

Since participants were required to schedule in equal numbers of enjoyable or routine activities, we repeated the analyses testing differences in activity ratings from pre to post activity scheduling separately for ratings of enjoyable vs. routine activities.

For enjoyable activities, there was a significant Condition x Time interaction for motivation, *F*(1,70) = 8.53, *p* = .005, η^2^ = 0.109, indicating that change in motivation to engage in enjoyable activities from pre to post activity scheduling differed between the two conditions. Motivation to engage thus increased in both conditions and this increase was relatively stronger in the motivational imagery condition, as predicted. The Condition x Time interaction for anticipated pleasure, anticipated reward and anticipated effort for enjoyable activities was not significant, indicating that for enjoyable activities, change in these variables from pre to post activity scheduling did not differ between the two conditions.

For routine activities, there was a significant Condition x Time interaction for anticipated pleasure, *F*(1,70) = 4.19, *p* = .044, η^2^ = 0.057 and anticipated reward, *F*(1,70) = 8.96, *p* = .004, η^2^ = 0.113, indicating that change in anticipated pleasure and anticipated reward of routine activities differed between the two conditions. Anticipated pleasure increased in both conditions and this increase was relatively stronger in the motivational imagery condition. Anticipated reward from engaging in routine activities increased in the motivational imagery condition whereas it remained stable in the combined control condition. The Condition x Time interaction for motivation and anticipated effort was not significant, indicating that for routine activities, change in these variables from pre to post activity scheduling did not differ between the two conditions.

*Relation between imagery vividness and activity ratings within the motivational imagery group separately for enjoyable and routine activities*

For enjoyable activities, there was a significant positive correlation between vividness ratings of enjoyable activities and motivation to engage in enjoyable activities (*r* = .42, *p* = .048), suggesting that the more vividly participants in the motivational imagery group imagined engaging in enjoyable activities, the more motivated they were to engage in these activities (or vice versa). Correlations between vividness ratings for enjoyable activities and anticipated pleasure, reward and effort of enjoyable activities were not significant.

For routine activities, there was a significant positive correlation between vividness ratings of routine activities and anticipated reward, *r* = .42, *p* = .045, suggesting that the more vividly participants in the motivational imagery condition imagined engaging in routine activities, the more anticipated reward from engaging in these activities they reported. Correlations between vividness ratings for routine activities and anticipated pleasure, effort and motivation of routine activities were not significant.

***Supplementary Behavioural activity outcomes***

*Effects of the motivational imagery task on the number of completed activities separately for enjoyable and routine activities*

There was a significant group difference in the mean number of completed routine activities, *F*(2,67) = 4.34, *p* = .017. Post-hoc comparisons with Bonferroni correction for multiple testing revealed a significant difference between the mean number of completed routine activities in the motivational imagery group (M = 2.39, SD = 0.72) and the activity reminder group (M = 1.70, SD = 0.82), *p* = .02, *d* = 0.89. There was no statistical significant difference between the mean number of completed routine activities in the motivational imagery condition and the no-imagery no-reminder control condition (M = 2.20, SD = 0.93), *p* =1.00, *d* = 0.23; There was no statistical significant difference in activity completion rate between the two control conditions, *p* =.11, *d* = 0.52;

The three groups did not differ in the mean number of completed enjoyable activities, *F*(2,66) = 0.25, *p* = .78.

*Mood and activity rating upon activity completion separately for enjoyable and routine activities*

On average, enjoyable activities were rated as pleasant (M = 77.05, SD = 13.64) and rewarding (M = 79.15, SD = 14.63) and as not taking a lot of effort (M = 39.33, SD = 16.75). Mood ratings during completion of enjoyable activities were high (M = 76.56, SD = 12.20). There were no differences in these ratings between the three groups (all p-values >.05).

On average, completed routine activities were rated as neither pleasant nor unpleasant (M= 54.75, SD = 19.42) and neither effortful nor not effortful (M = 53.51, SD = 21.20). Completed routine activities were rated as rewarding (M = 76.71, SD = 13.04) and participants reported positive mood on average during completion of routine activities (M = 65.39, SD = 17.46). There were no differences in these ratings between groups (all p-values >.05).

**Supplementary Table 1** Activity list

| Spend some time viewing photos or scrapbook |
| --- |
| Do some meditation / relaxation |
| Play cards / board game / computer game - on own or with someone |
| Learn / practise a language or other skill |
| Read a book / magazine / newspaper |
| Engage in something creative: scrapbooking / craft / editing photos / drawing / colouring in / painting |
| Soak in the bath |
| Listen to the radio / music / favourite podcast |
| Read before bed |
| Walk or play with the dog or other pet |
| Cook or bake something or perhaps try a new recipe |
| Exercise / fitness / go to the gym / play tennis or basketball etc. |
| Ring a family member / friend / auntie / grandma on the phone, maybe for their birthday |
| Do some yoga / pilates / stretches |
| Talk to / spend time with a family member / friend in person |
| Sing / dance / play an instrument |
| Work on a crossword / puzzle / Sudoku |
| Have a nap / rest |
| Go for a walk / run / bikeride / drive in the countryside |
| Self care: polishing nails, styling hair, medications, eyecare, etc. |
| Enjoy a hot drink / favourite snack / meal out / lunch / dinner |
| Clean / tidy / organise at home, e.g. hoover or clean a room or organise a cupboard/desk drawer |
| Run errands outside the home e.g. trip to shop or drycleaners or locksmith |
| Sort household paperwork |
| Take care of correspondence e.g. birthday card or email or letter to a friend |
| Planning and decision-making e.g. planning a weekend away or a special birthday |
| DIY / house repairs, e.g fix a broken chair or hang pictures |
| Install and/or learn to use new software |
| Give something a good clean: stove, fridge, kitchen floor, shower, kitchen floor, etc. |
| Tend to financial matters: paying bills / switching providers |
| Clear out old items / take to charity shop |
| Care for plants (house or garden), e.g. water or weed or mow the lawn |
| Mend or iron clothes |
| Care for pet e.g. bathe the dog |
| Research how to do something e.g. bicycle repair |
| Resume work on a project that has been on hold |
| Go to bed early to get enough sleep |
| Send birthday / thank you / invitation cards |
| Tend to 'business' emails or phone calls you have been putting off, e.g. email or phone clients |
| Bicycle maintenance or repair e.g. clean, pump or change tires, etc. |
| Plan a holiday |
| Maintenance of computers, mobile phones, backing up etc. |
| Play computer games |
| Diary/journal entry |
| Complete homework |
| Tend to kit for a hobby, e.g. organise fishing equipment, clean rugby shoes, organise sewing box |
| Start an essay |
| Do extra reading for your course/essay |
| Write up lecture notes |
| Take a lunch break away from desk |
